# A metagenomic study of gut viral markers in amyloid-positive Alzheimer’s disease patients

**DOI:** 10.1186/s13195-023-01285-8

**Published:** 2023-08-22

**Authors:** Mahin Ghorbani, Daniel Ferreira, Silvia Maioli

**Affiliations:** 1 Department of Dental Medicine, Karolinska Institute, Stockholm, Sweden; 2 Division of Clinical Geriatrics, Center for Alzheimer Research, Department of Neurobiology, Care Sciences and Society, Karolinska Institutet, Stockholm, Sweden; 3 Facultad de Ciencias de la Salud, Universidad Fernando Pessoa Canarias, Las Palmas, España; 4 Division of Neurogeriatrics, Center for Alzheimer Research, Department of Neurobiology, Care Sciences and Society, Karolinska Institutet, Stockholm, Sweden

**Keywords:** Gut virome, Alzheimer’s disease, Amyloid-β, Bacteriophage, Whole-genome sequencing

## Abstract

**Background:**

Mounting evidence suggests the involvement of viruses in the development and treatment of Alzheimer’s disease (AD). However, there remains a significant research gap in metagenomic studies investigating the gut virome of AD patients, leaving gut viral dysbiosis in AD unexplored. This study aimed to fill this gap by conducting a metagenomics analysis of the gut virome in both amyloid-positive AD patients (Aβ + ADs) and healthy controls (HCs), with the objective of identifying viral signatures linked with AD.

**Method:**

Whole-genome sequence (WGS) data from 65 human participants, including 30 Aβ + ADs and 35 HCs, was obtained from the database NCBI SRA (Bio Project: PRJEB47976). The Metaphlan3 pipeline and linear discriminant analysis effect size (LEfSe) analysis were utilized for the bioinformatics process and the detection of viral signatures, respectively. In addition, the Benjamini–Hochberg method was applied with a significance cutoff of 0.05 to evaluate the false discovery rate for all biomarkers identified by LEfSe. The CombiROC model was employed to determine the discriminatory power of the viral signatures identified by LEfSe.

**Results:**

Compared to HCs, the gut virome profiles of Aβ + ADs showed lower alpha diversity, indicating a lower bacteriophage richness. The *Siphoviridae* family was decreased in Aβ + ADs. Significant decreases of *Lactococcus phages* were found in Aβ + ADs, including *bIL285*, *Lactococcus phage bIL286*, *Lactococcus phage bIL309*, and *Lactococcus phage BK5 T*, *Lactococcus phage BM13*, *Lactococcus phage P335 *sensu lato, *Lactococcus phage phiLC3*, *Lactococcus phage r1t*, *Lactococcus phage Tuc2009*, *Lactococcus phage ul36*, and *Lactococcus virus bIL67*. The predictive combined model of these viral signatures obtained an area under the curve of 0.958 when discriminating Aβ + ADs from HCs.

**Conclusion:**

This is the first study to identify distinct viral signatures in the intestine that can be used to effectively distinguish individuals with AD from HCs.

**Supplementary Information:**

The online version contains supplementary material available at 10.1186/s13195-023-01285-8.

## Background

Alzheimer’s disease (AD), which affects approximately 50 million people globally, is the leading cause of dementia and a serious global health concern [1]. The disease is characterized by the gradual accumulation of amyloid β-protein (Aβ) plaque and tangles of hyperphosphorylated tau neurofibrils, which result in neuroinflammation, neuronal loss, and cognitive impairment [2, 3]. Despite numerous hypotheses proposed to explain the pathogenesis and progression of AD, the precise causes of its onset and course remain unknown. Consequently, although several therapeutic approaches have been suggested, many clinical trials have failed to provide significant benefits [4, 5]. However, the Food and Drug Administration (FDA) recently approved lecanemab [6, 7] and aducanumab [8], which are monoclonal antibodies that target and remove beta-amyloid plaques in the brain. While these are positive developments, the need for effective AD treatments remains urgent. Researchers continue their quest to identify potential biomarkers for early disease detection so that treatment is initiated before the disease has progressed. However, the translation of these findings into clinical practice is still a long way off [9].

In recent years, there has been an explosion of interest in the gut microbiota-brain axis, which has been suggested as a therapeutic target for central nervous system illnesses [10]. A growing body of clinical and pre-clinical research points to the gut microbiome as a key player in the regulation of neurodegenerative processes, cognitive functions, and neurological disorders including AD [11–13]. Most studies rely on bacterial components of the gut microbiome rather than viral components. Furthermore, growing evidence supports the role of viruses in the etiology of AD, as well as the interaction between viral and bacterial components of the gut microbiome [14–17]. However, there is a lack of metagenomics research profiling the gut virome of AD patients, which could reveal unexplored gut viral “dark matter” in AD. The term human virome is used to describe the human microbiome’s viral components. The human virome, also called the viral metagenome, is the complete set of viruses found in humans. The virome comprised around 10^13^ particles per human and has a high level of heterogeneity. The human virome contains both eukaryotic and prokaryotic viruses including bacteriophages [18–20]. The effects of eukaryotic viruses on human health are wide-ranging, from those that cause very mild, self-limiting acute or chronic infections to those that have catastrophic outcomes [19, 21]. Bacteriophages, the most abundant member of the human virome, play an important role in the constant regulation of the diversity, richness, abundance, evolution, and physiology of microbial communities, so they may play an important role in human health by controlling bacteriome balance [22, 23]. Some examples of bacteriophage under research and potential therapy are *Escherichia virus T4*, which is well-studied and used in research that infects *E. coli* and shows potential for phage therapy [24]. *PhiX174 phage* is another bacteriophage that infects enteric bacteria like *E. coli*, serving as a valuable model organism in molecular biology research [25]. *Pseudomonas phage* targets and kill *Pseudomonas* bacteria, offering alternative treatment options for infections [26]. Bacteriophage *ICP1* is a potential therapeutics against Vibrio-related infections like *cholera* [27]. These four bacteriophages demonstrate the diverse applications and potential benefits of potential phage therapy and molecular biology research. Continued research into bacteriophages may lead to innovative approaches for combating bacterial infections and maintaining a healthy bacteriome.

Bacteriome dysbiosis is believed to be an etiologic factor in the development of a number of diseases, including AD [13, 28]. Depending on the viral type, it is considered that viruses have a dual role in AD. Eukaryotic viruses have been discovered as probable causes of dementia, whereas some bacteriophages are potential therapeutic agents [29, 30]. Growing evidence links the *Herpesviridae* virus family, specifically HSV-1, to the etiology of AD. HSV-1 DNA has been detected within amyloid plaques located in the temporal and frontal cortices of individuals with AD. While several studies have indicated a higher prevalence of HSV-1 in AD patients, it is important to note that the exact pathogenic mechanisms remain unclear. It is believed that HSV-1 may not act as a sole causal agent in AD but rather its interactions with the host could contribute to the underlying pathophysiology of the disease [29, 31, 32]. Further research is needed to fully understand the intricate relationship between HSV-1 and the development of AD.

A previous study demonstrated the association of bacteriophages with improved executive function and verbal memory in flies, mice, and humans [30]. Through their research, the authors observed that individuals with elevated levels of *Caudovirales* and *Siphoviridae* in their gut virome exhibited enhanced executive function and verbal memory, as evidenced by both a discovery cohort (*n* = 114) and a validation cohort (*n* = 942). Conversely, heightened levels of *Microviridae* were linked to more severe impairments in executive functions. In an experiment involving mice, those receiving a virome transplant from humans with high levels of a specific *Caudovirales* (> 90% from the Siphoviridae family) performed better on a novel object recognition test, accompanied by increased expression of immediate early genes involved in memory formation in the prefrontal cortex. Additionally, the introduction of the 936 group of *lactococcal Siphoviridae* bacteriophages to the diet of *Drosophila* flies improved memory scores and upregulated memory-related brain genes. As a result, bacteriophages hold promise as potential actors within the virome-brain axis and warrant further investigation.

The current understanding of the connection between viruses and AD is supported by a substantial body of evidence [33–35]. However, there remains a notable research gap concerning the specific viral components present in the gut microbiome of AD patients. To address this knowledge deficiency, in the present study, we conducted a metagenomics analysis with the objective of identifying gut viral signatures that can effectively distinguish individuals with AD from healthy controls (HCs).

## Methods and materials

### Data collection

For our study, we utilized raw fecal sample data from the NCBI SRA database under the BioProject number PRJEB47976. This reference BioProject specifically focused on studying the gut bacteriome in two groups: Aβ + ADs (*n* = 75) and HCs (*n* = 100). The inclusion criteria for Aβ + ADs required them to meet the NIA-AA core clinical criteria for probable AD dementia, with a global clinical dementia rating (CDR) score ranging from 0.5 to 1.0. They also needed to exhibit evidence of cerebral amyloid β (Aβ) accumulation, as indicated by cerebrospinal fluid (CSF) levels of Aβ42 below 600 pg/ml. HCs were selected based on the absence of neurological or psychiatric disorders, no history of cognitive decline, and having a global CDR score of 0 and an MMSE score of 27 or higher. The objective of BioProject number PRJEB47976 was to explore the relationship between gut bacteriome signatures discriminating Aβ + ADs from HCs, where metagenomic sequencing analysis was used to assess the gut bacteriome, leading to the identification of 18 specific bacterial genera that showed significant associations with AD [36]. The combined contribution of these 18 genera was found to be linked to the presence and progression of AD, offering potential avenues for improvement. Since age and gender have been found to have a relevant effect on the gut microbiome [37–39], for our current study, we subsampled the reference study [36]. Initially, we selected 35 samples for each group. However, during the bioinformatics analysis, we had to exclude 5 samples due to their very low viral library sample size from the Aβ + AD group. This exclusion was crucial to ensure the reliability of the results, as low library size could potentially impact the accuracy of our findings, possibly resulting from improper DNA degradation or extraction for viral agents. In the end, we proceeded with a total of 65 samples, consisting of 30 individuals with Aβ + AD and 35 HCs. Despite the exclusion of 5 samples, our final subsampled cohort did not exhibit any statistically significant group differences in terms of age or gender.

In this study, a power analysis was conducted to determine the appropriate sample size for comparing two groups (Aβ + ADs and HCs) using Hedges’ *g* and Cohen’s *d* effect size measures. The significance level was set at 0.05 to control false positives, and a statistical power of 80% was aimed to detect true effects effectively. With a moderate effect size of 0.66, the calculated sample size of 30 Aβ + ADs and 35 HCs was considered sufficient to achieve the desired statistical power and detect meaningful differences between the groups. Differently from the reference study [36] that investigated the gut bacteriome, our specific focus in the current study was on investigating the gut viral signatures present in these samples, expanding the understanding of the gut virome in relation to AD. Supplementary Table S1 contains information about the selected samples including NCBI accession, age, gender, reads length (bp), and base pairs.

### Bioinformatics and statistical analysis

Raw RNA-Seq data were quality-checked using FastQC v0.11.8 before being pre-processed with Cutadapt v2.8 to remove adapter sequences and low-quality bases. MetaPhlAn3 [40] was used to process the pre-processed sequencing reads, which rely on unique clade-specific marker genes identified from 17,000 reference genomes (13,500 bacterial and archaeal, 3500 viral, and 110 eukaryotic). The functions —ignore bacteria, —ignore eukaryotes, and —ignore archaea were used to exclude bacterial, eukaryotic, and archaeal taxa. The internal Metaphlan3 database was used to make taxonomic assignments. The feature count table was filtered to exclude counts < 2 with a minimum sample prevalence of 10%. The final feature count table for downstream analysis was generated using total sum of scales (TSS) normalization followed by rarefication for sample depth normalization.

Alpha diversity metrics such as observed, Shannon, Simpson, and differential viral communities (beta diversity) between Aβ + ADs and HCs were analyzed with the vegan package v2.5.6 in the R environment utilizing Bray–Curtis-based non-metric multidimensional scaling (NMDS) with the PERMANOVA significance test. Linear discriminant analysis effect size (LEfSe v1.1.01) (http://huttenhower.sph.harvard.edu/galaxy) (LDA > 2.00, and *p* < 0.05) was used to identify differentially abundant species between Aβ + ADs and HCs [41]. The area under the receiver operating characteristic (AUROC) analysis was used to determine the predictive value of each identified viral species, and the CombiROC tool [42] was used to select accurate viral feature combinations. MATLAB was used to visualize the final ROC curve.

## Results

### Study design and participants

We analyzed a total of 65 samples from 30 Aβ + ADs and 35 HCs. In our subsampled cohort, there were no statistically significant group differences in age or gender among the participants, as we subsampled them based on these factors. Additionally, the reference study [36] reported no statistically significant differences between Aβ + AD and HCs in body mass index, arterial hypertension, diabetes mellitus, and rheumatoid arthritis status, while there were statistically significant differences in cognitive function (MMSE) with reduced performance in the Aβ + AD and higher usage of AChE inhibitors and antidepressants in Aβ + AD, as expected [36].

**Fig. 1:**
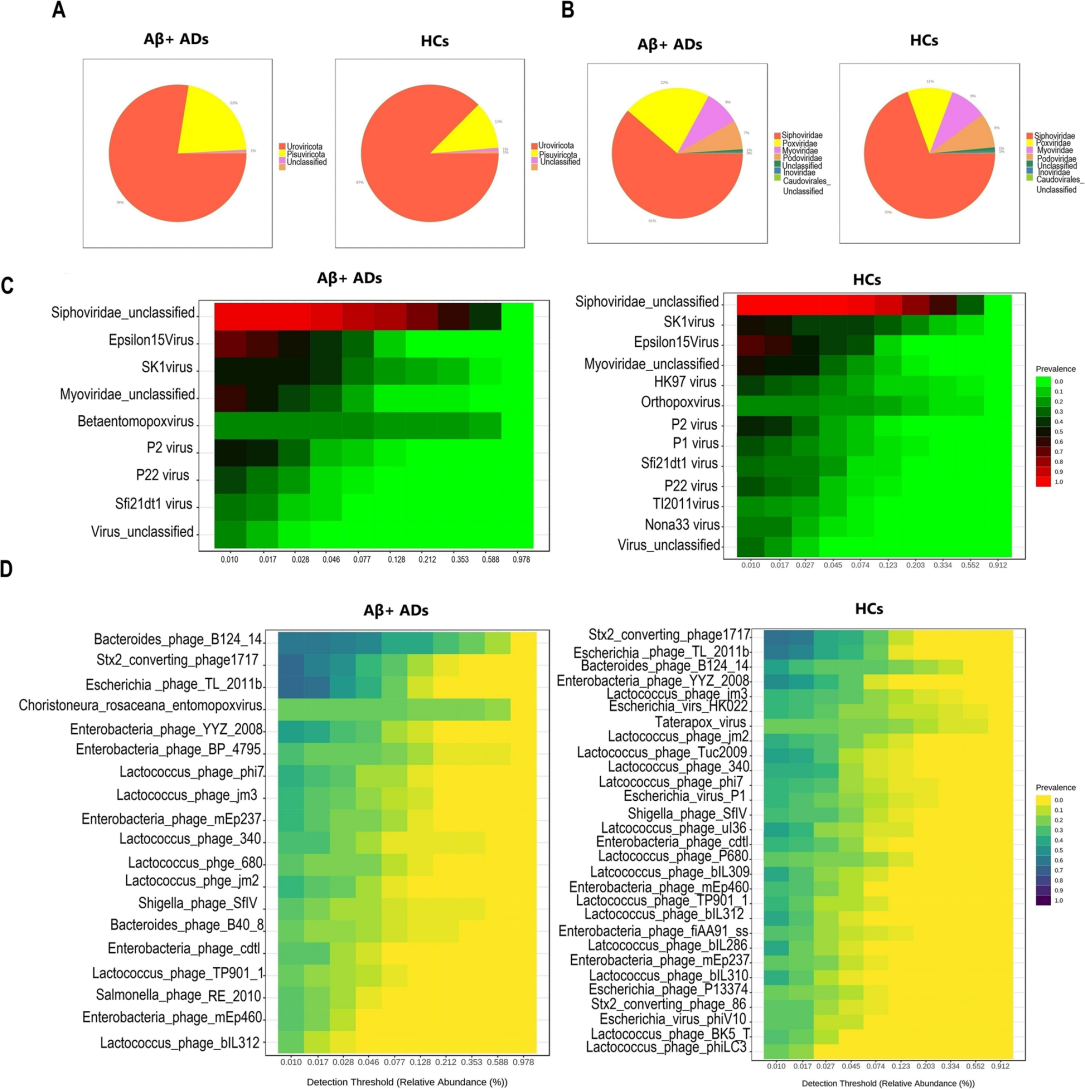
Gut virome composition profiles and core gut virome of Aβ + ADs and HCs. **A**, **B** Pie charts depicting the viral distribution in Aβ + AD individuals and HCs at the phylum and family levels, respectively. **C**, **D** Heatmaps of the core virome in Aβ + ADs and HCs at the genus and species levels, respectively

In our subsample, the total number of sequence reads obtained from 65 samples was 2,267,731,716, with a range of 26,776,840 to 46,799,316 and an average of 34,888,180. The total number of feature reads from all samples was 1,474,732, with an average of 22,688 OUT per sample and a range of 1124 to 421,766. Following 10% prevalence and 2 > filtration, eighty viruses were obtained and assigned to four phyla, seven families, twenty-two genera, and eighty species. All samples have Good’s coverage of more than 99%. At the phylum level, an interactive pie chart shows that *Hofneiviricota*, *Pisuviricota*, and *Uroviricota* were present in both groups, with *Uroviricota* being the most prevalent (Aβ + ADs: 78%, HCs: 87%). In Aβ + ADs, the abundance of *Uroviricota* is 9% lower than in HCs, while the abundance of *Pisuvirico* is 11% higher (Fig. 1A).

At the family level, an interactive pie chart reveals that *Siphoviridae*, *Poxviridae*, *Myoviridae*, and *Podoviridae* are the most abundant families in both groups. Comparing Aβ + ADs to HCs, the abundance of *Siphoviridae* and *Podoviridae* is reduced by 9% and 2%, respectively, whereas the abundance of *Poxviridae* is increased by 11% (Fig. 1B). Core virome at the genus and species levels was set for 20% prevalence of samples in each group and minimum 0.01% abundance. The core virome of the Aβ + ADs consisted of 9 genera and 19 species, whereas the core virome of HCs consisted of 19 and 29 viral features. The most prevalent genera in both groups were *Siphoviridae unclassified*, *Epsilon15virus*, *Myoviridae unclassified*, and *Sk1viru*. *Betaenttomopoxvirus* was only found in core virome of Aβ + ADs, whereas *Hk97virus*, *Orthopoxvirus*, *Tl2011virus*, and *Nona33virus* were found only in core virome of the HCs based on the setting scores (Fig. 1C). The heatmaps of the dominant core virome at the species level show that *Enterobacteria phage BP 4795*, *Salmonella phage RE 2010*, *Choristoneura rosaceana entomopoxvirus*, and *Bacteroides phage B40 8* were only found in Aβ + ADs, whereas 14 species, including *Lactococcus phage ul36*, *Lactococcus phage Tuc2009*, *Lactococcus phage bIL309*, *Lactococcus phage bIL286*, *Lactococcus phage bIL310*, *Escherichia virus P1*, *Escherichia virus HK022*, *Lactococcus phage BK5 T*, *Enterobacteria phage fiAA91 ss*, *Stx2 converting phage 86*, *Escherichia virus phiV10*, *Taterapox virus*, *Lactococcus phage phiLC3*, and *Escherichia phage P13374*, were exclusive in core virome of HCs under conditions of 20% sample prevalence and > 0.01 abundance (Fig. 1D).

### Virome richness and diversity in participants’ guts

**Fig. 2:**
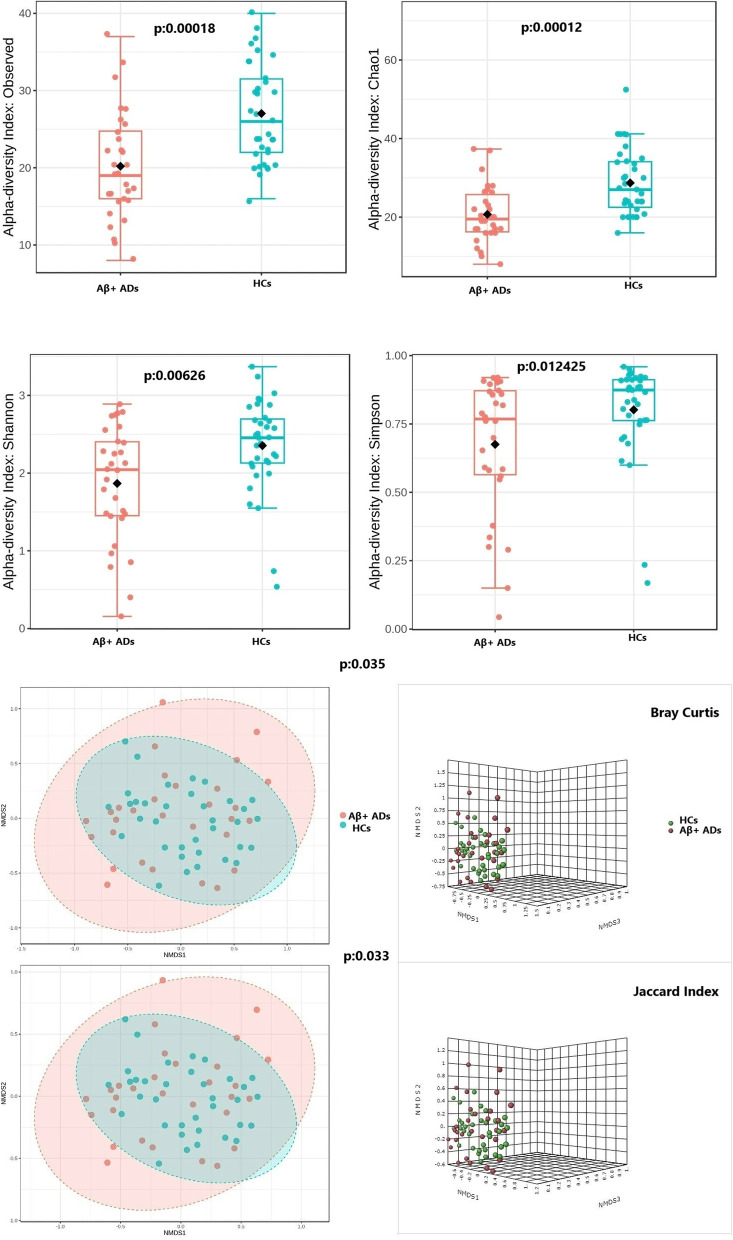
Gut virome richness, diversity, and interpersonal variations in Aβ + ADs and HCs. **A** Boxplots displaying the alpha diversity measurements, including the observed and Chao1, Shannon, and Simpson’s indices between Aβ + ADs and HCs. **B** Beta diversity measurements based on NMDS using Bray–Curtis dissimilarity distances and the Jaccard index with PERMANOVA test assessment between Aβ + ADs and HCs

Compared to HCs, Aβ + ADs exhibited lower alpha diversity in their gut virome profiles, as evidenced by the results of various diversity measures (observed index: *p* < 0.0001; Chao1 index: *p* < 0.0001; Shannon index: *p* = 0.0062; Simpson’s index: *p* = 0.0124) (Fig. 2A). Additionally, the analysis based on NMDS using Bray–Curtis dissimilarity distances and Jaccard index revealed notable interpersonal variations between Aβ + ADs and HCs (Bray–Curtis and PERMANOVA: *p* = 0.035; Jaccard index PERMANOVA: *p* = 0.033) (Fig. 2B).

### *Taxonomic differences of gut virome in Aβ* + *ADs and HCs*

**Fig. 3:**
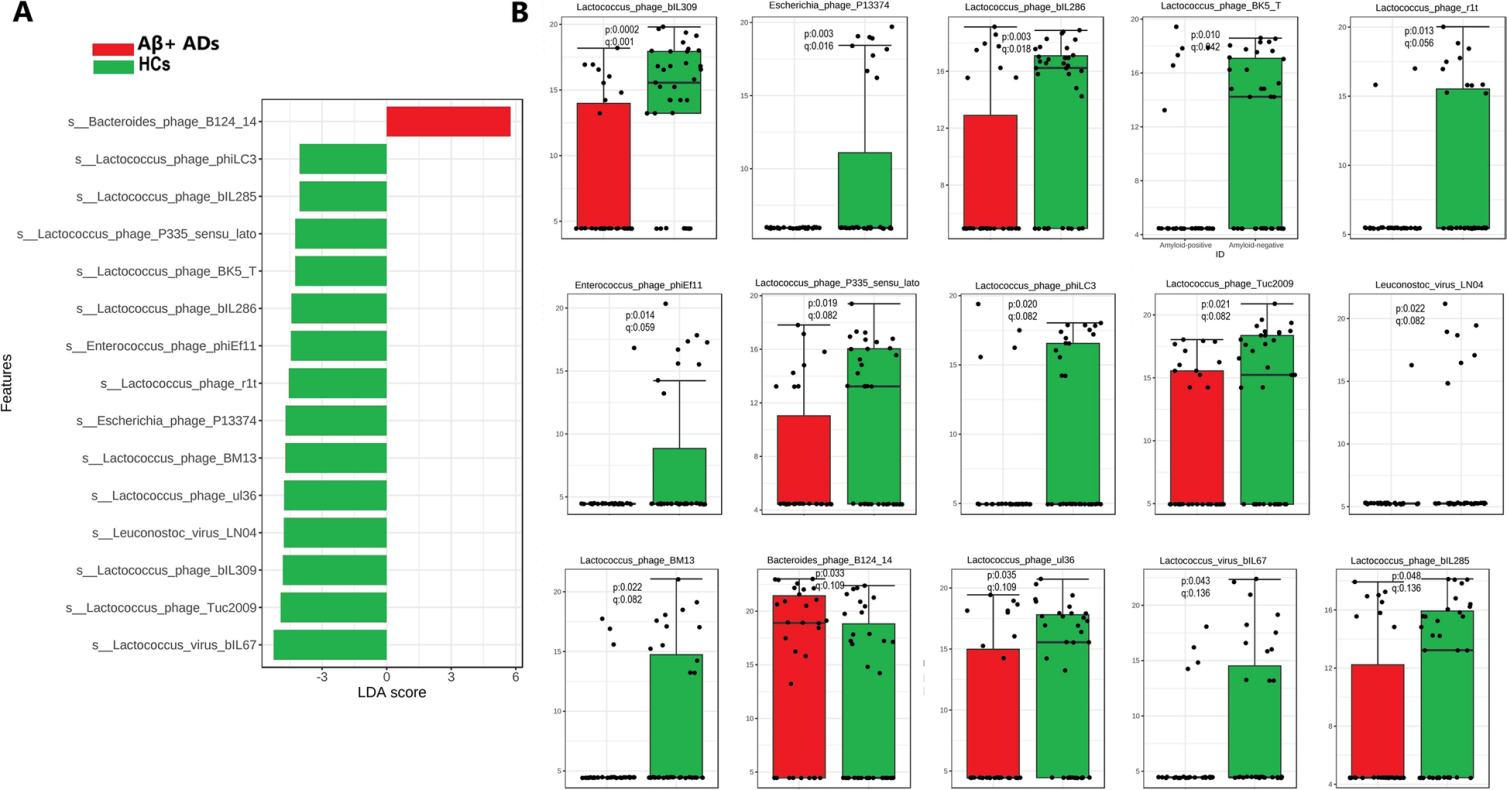
Gut viral signatures significantly different between Aβ + ADs and HCs. **A** LEfSe analysis identified the most differentially abundant viral species in Aβ + ADs and HCs (*p* < .05; LDA score 2), viral species enriched in HCs are indicated with negative LDA scores (green), and viral species enriched in Aβ + ADs are indicated with positive LDA scores (red color). **B** Pairwise Wilcoxon test for each significant viral species identified by LEfSe analysis (*p* < 0.05) and Benjamini–Hochberg (BH) test, for false discovery rate (FDR) adjustment (*q* < 0.05)

LEfSe analysis was used to identify viral species that were differentially abundant between Aβ + ADs and HCs. Figure 3A shows that, based on LDA scores, compared to HCs, Aβ + ADs exhibited a significant increase in the abundance of *Bacteroides phage B124* and a significant decrease in the abundance of several *Lactococcus phages*, including *bIL285*, *Lactococcus phage bIL286*, *Lactococcus phage bIL309*, *Lactococcus phage BK5 T*, *Lactococcus phage BM13*, *Lactococcus phage P335 *sensu lato, *Lactococcus phage phiLC3*, *Lactococcus phage r1t*, *Lactococcus phage Tuc2009*, *Lactococcus phage ul36*, and *Lactococcus virus bIL67*. Additionally, Aβ + ADs also exhibited reduced levels of *Escherichia phage P13374* and *Leuconostoc virus LN* when compared to HCs. Pairwise Wilcoxon test for viral signatures between Aβ + ADs and HCs by LEfSe analysis (*p* < 0.05) is shown in (Fig. 3B).

We conducted the Benjamini–Hochberg (BH) test, a false discovery rate (FDR) controlling procedure, at a significance level of *q* < 0.05. As a result, *Lactococcus phagebIL309*, *Escherichia phage P13374*, *Lactococcus phage bIL286*, *Lactococcus*, and *phage BK5 T* achieved *q* values below 0.05, indicating significant findings. However, *Lactococcus phage r1t*, *Enterococcus phage phiEf11*, *Lactococcus phage P335 *sensu lato, *Lactococcus phage phiLC3*, *Lactococcus phage Tuc2009*, *Leuconostoc virus LN04*, and *Lactococcus phage BM13* obtained *q* values ranging from 0.056 to 0.082, and the remaining four viral signatures obtained *q* values above 0.1, suggesting non-significant results when adjusting for multiple comparisons (Fig. 3B).

**Fig. 4:**
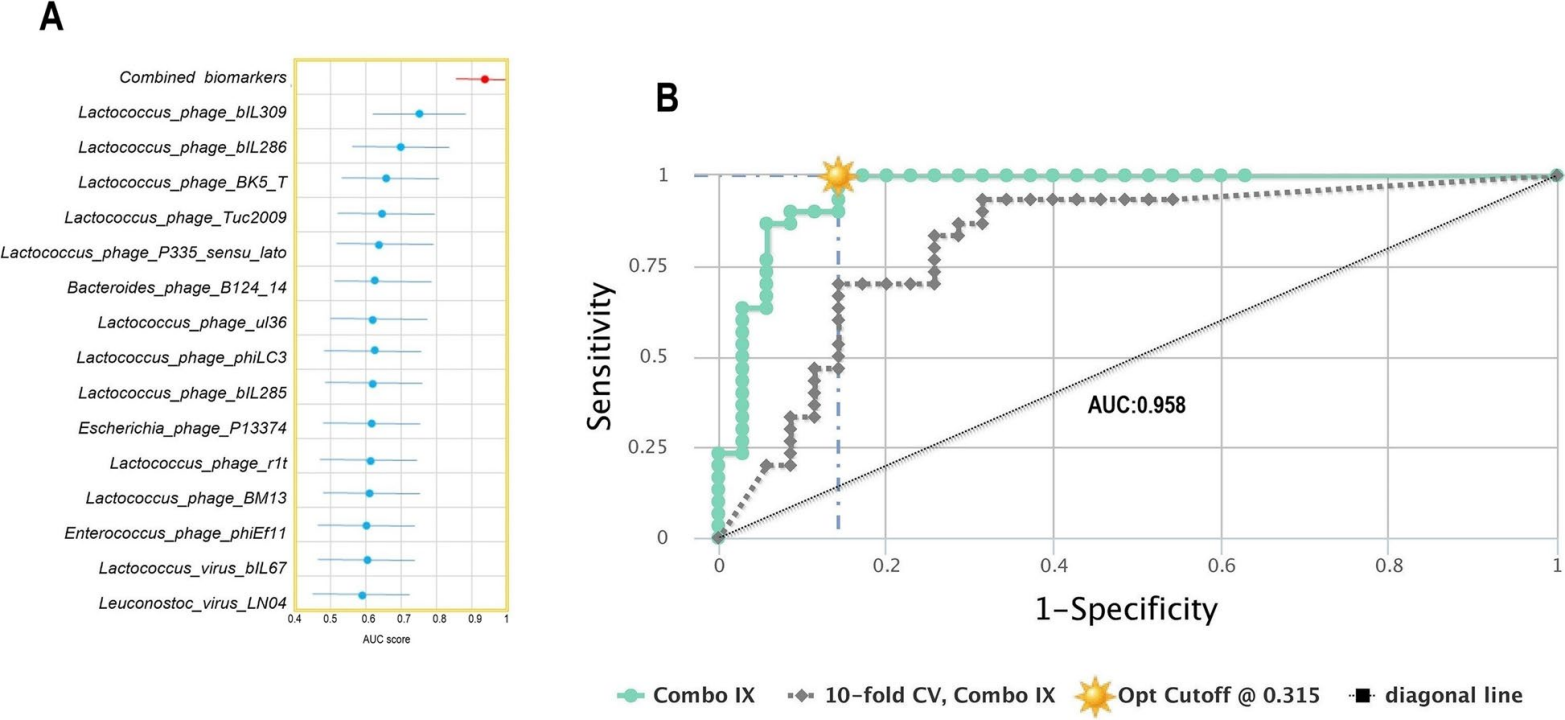
Area under the receiver operating characteristic (AUROC) analysis of viral signatures for discriminating Aβ + ADs and HCs. **A** The area under the curve scores show the predictive values of the individual viral signature and the combined viral signatures. **B** Combined model graphical representation of viral signatures

The ability power of the identified viral signatures to discriminate between individuals with Aβ + ADs and HCs was evaluated using AUROC analysis. This analysis aimed to assess the strength of these viral signatures as predictive classifiers for distinguishing between the two groups. The AUROC results indicated that viral signatures such as *Lactococcus phage bIL309*, *Lactococcus phage bIL286*, *Lactococcus phage BK5 T*, *Lactococcus phage P335 *sensu lato, and *Bacteroides phage B124 14* provided a predictive value of 0.64 to 0.75 (AUROC; 95% CI, *p* < 0.05) whereas the remaining nine features exhibited AUROC values ranging from 0.60 to 0.64 with *p*-values ranging from 0.054 to 0.169 (AUROC; 95% CI) (Fig. 4A). Combining all fifteen statistically significant viral characteristics increased the AUROC score to 0.96% (*p* < 0.0001) (Fig. 4A).

## Discussion

This study investigated the composition of the gut virome in a cohort of Aβ + AD patients and HCs. The gut virome profiles of Aβ + ADs revealed reduced alpha diversity as compared to HCs, which suggested a lower bacteriophage richness. Our analyses showed that *Uroviricota* was the most frequent phylum of *Caudovirales* bacteriophages in both groups, and that was reduced by 9% in Aβ + ADs compared to HCs. The abundance of the family *Siphoviridae* was reduced by 9% in Aβ + ADs compared to HCs, whereas the abundance of the family *Poxviridae* was increased by 11%. A previous study discovered that *Caudovirales bacteriophages* are associated with improved executive function and memory in flies, mice, and humans [30]. The study demonstrated that the levels of various *Caudovirales*, specifically the *Siphoviridae* family, were negatively correlated with the Trail Making Test (TMT) score, where a longer time taken to complete the test is generally associated with poorer performance or cognitive impairment, whereas the levels of *Microviridae* were positively correlated with the TMT score. Also, the same study discovered choline and glycine, the two most prominent sources of 1C units of folate, had the strongest relationships with *Microviridae* and *Caudovirales* levels [30]. Choline blocks the production of amyloid-beta plaques [43] and also choline supplementation reduces amyloidosis and increases choline acetyltransferase expression in the hippocampus of APPswePS1dE9 AD model mice [44]. A significant correlation exists between the degree of choline acetyltransferase activity loss in cerebral cholinergic neurons and the severity of dementia or cognitive deficits reported in AD [45]. Our LEfSe analysis revealed that Aβ + ADs significantly had a lower abundance of several *Lactococcus phages* including *bIL285*, *Lactococcus phage bIL286*, *Lactococcus phage bIL309*, *Lactococcus phage BK5 T*, *Lactococcus phage BM13*, *Lactococcus phage P335 *sensu lato, *Lactococcus phage phiLC3*, *Lactococcus phage r1t*, *Lactococcus phage Tuc2009*, *Lactococcus phage ul36*, and *Lactococcus virus bIL67*. *Lactococcus* phages belong to the order *Caudovirales* and have double-stranded DNA (dsDNA) genome. They are one of the most common phages that infect bacteria, particularly *Lactococcus* species [46]. Interaction between *Lactococcus* phage and *Lactococcus* bacterial species in AD should be examined, as bacteriophages regulate the diversity of *Lactococcus* bacteria and modulate their metabolic pathways. *Lactococcus*, a genus of lactic acid bacteria (LAB), is known for its production of lactic acid. Within humans, there exist two forms of lactic acid: l-lactic acid and d-lactic acid. While l-lactic acid is a commonly found compound in human metabolism, d-lactic acid is primarily produced by specific microorganism strains or less significant metabolic pathways. Despite their distinct structures, effects on the human body, and mechanisms of action, most studies do not differentiate between these two forms [47–49]. The production of lactic acid occurs through diverse metabolic pathways. l-lactic acid arises from the catabolism of amino acids and carbohydrates during glycolysis, whereas d-lactic acid is generated from the metabolism of carbohydrates, lipids, and intestinal bacteria. Both l-lactic acid and d-lactic acid impact neural network activity by binding to the hydroxycarboxylic acid receptor 1. l-lactic acid plays a crucial role in neural oxidative metabolism, contributing to memory formation, protein synthesis, synaptic remodeling, and axonal excitability. Conversely, d-lactic acid can hinder the uptake of l-lactic acid by neurons, resulting in inadequate neuronal energy metabolism and memory impairment [50–53].

Memory function relies on lactic acid [54, 55]. A previous study has shown that astrocytes in AD patients secrete less lactic acid, potentially contributing to the pathogenesis of the disease [56]. Neuronal energy metabolism depends on lactic acid produced by astrocytes [57, 58]. Lactic acid generated through glycolysis serves as an energy source for the brain and protects neurons from mitochondrial damage caused by Aβ protein accumulation [53]. Lactic acid appears to play a bidirectional role in AD etiology. On the one hand, long-term memory requires lactic acid from astrocytes to neurons. On the other hand, the accumulation of lactic acid in the brain stimulates Aβ protein deposition and excessive transmission of lactic acid into neurons, leading to lower pH, mitochondrial dysfunction, apoptosis, and impaired brain function [53–55]. Therefore, the regulation of gut bacteria and their lactic acid products by bacteriophage in AD requires further investigation. Our findings provide valuable insights into the role of gut dysbiosis in AD and align with a recent study that proposes a theoretical framework and hypothesis about the gut-brain axis and the role of gut microbiota in AD [59].

This study has several limitations that should be considered. The SRA database provided limited information on the clinical characteristics of the subjects, which restricted our ability to analyze associations between the gut virome and clinical features. We acknowledge the limitation of not having access to data on *APOE* genotype, diet, and medication use such as antibiotic and antidepressant use, which could influence gut virome diversity in our study. We recommend that further studies focus on exploring the impact of these variables on the gut virome to gain a more comprehensive understanding. Moreover, applying propensity scores on all these variables could offer an approach to match profiles between the two compared groups. Additionally, the sample size was relatively small. In future studies, maintaining a sufficient sample size above the required confidence level is imperative, particularly when planning further analysis post-study design. To prevent the exclusion of samples based solely on low viral library size, rigorous quality controls should be implemented during sample collection and RNA extraction. This approach will address the problem of low library size and reduce the need for further sample exclusions, ultimately enhancing the scientific robustness of studies. It is important to note that this study had a cross-sectional design, making it difficult to establish a cause-and-effect relationship between the gut virome. Additionally, cerebrospinal fluid biomarkers were not available for HCs, and thus, the present study recognizes a potential limitation of including AB + individuals in the HC group, some of whom might have altered microbiomes. Although this may have led our study to underestimate the differences between AB + ADs and HCs, the reported significant differences are valid and informative. To gain deeper insights, we recommend conducting longitudinal studies on individuals with preclinical AD and monitoring the changes in their gut microbiomes over time. Also, our cross-sectional study faces challenges in determining the causality between diet, gut dysbiosis, and AD. The impact of memory loss in individuals with AD may further hinder their ability to maintain a healthy diet leading to potential issues with forgetfulness related to eating their food. For more accurate insights, future research should prioritize longitudinal or caregiver-observed studies to mitigate these issues and better understand the complex interactions involved. We employed a combined model of AUROC analysis to assess the predictive power of all discriminatory biomarkers identified as a cohesive set. We acknowledge that the combined model may be influenced by preselected viral factors that were significant in distinguishing the groups initially. This could lead to higher AUROC values in that analysis and to ensure the generalizability of our finding, we recommend further validation in an independent (confirmatory) cohort in future studies. Another limitation is that the study did not measure metabolites in the collected gut samples and incorporating metabolomic analyses would provide a more comprehensive understanding of the gut virome’s role in AD. Additionally, simultaneous investigation of the bacteriome and virome, along with exploring their interactions, could offer valuable insights into the underlying mechanisms of AD pathogenesis.

## Conclusion

In conclusion, this study suggests valuable new insights into the dysbiosis of the gut virome in Aβ + AD individuals compared to HCs. Through our metagenomic analyses, we have identified and characterized significant gut viral signatures that have the potential to serve as biomarkers for AD. These findings contribute to the understanding of the role of the gut virome in AD pathogenesis and may pave the way for the development of diagnostic or prognostic tools for AD based on the assessment of these viral signatures. Further research and validation studies are warranted to fully establish the clinical utility of these biomarkers in AD diagnosis and management.

### Supplementary Information

**Additional file 1: Supplemental Table S1.** Information about the selected samples.

**Additional file 2: Supplemental Fig. S1.** Sequence sample size.

## Data Availability

The metagenomic data are available under NCBI, SRA, Bio Project no. PRJEB47976. Permission based on free and unrestricted access to all of the data under Nucleotide Sequence Database Policies Science 298 [5597]: 1333 15 Nov 2002.

## Abbreviations

AD: Alzheimer’s disease
Aβ: Amyloid β-protein
AChE: Acetylcholinesterase
AUROC: Area under the receiver operating characteristic
BMI: Body mass index
CSF: Cerebrospinal fluid
dsDNA: Double-stranded DNA
FDA: Food and Drug Administration
HCs: Healthy controls
HSV-1: Herpes simplex virus 1
LDA: Linear discriminant analysis
MMSE: Mini-Mental State Examination
NCBI: National Center for Biotechnology Information
NMDS: Non-metric multidimensional scaling
PRJEB: BioProject identifier
SRA: Sequence Read Archive
TMT: Trail Making Test
WGS: Whole Genome Sequencing
