# Supplementary figures and images for: A metagenomic study of gut viral markers in amyloid-positive Alzheimer’s disease patients
Source: Alzheimers Res Ther. 2023 Aug 22;15:141. doi: 10.1186/s13195-023-01285-8 (PMC10464408; doi:10.1186/s13195-023-01285-8)

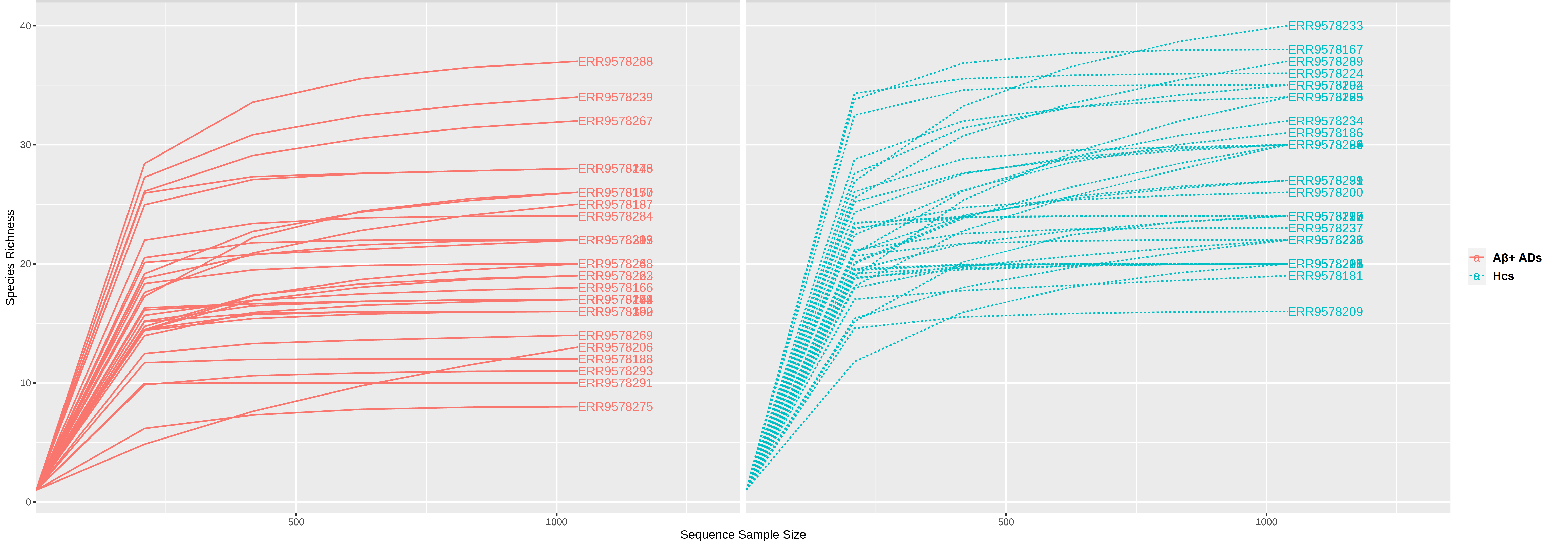

Supplement: Supplementary file 2 — Additional file 2: Supplemental Fig. S1. Sequence sample size. [file 13195_2023_1285_MOESM2_ESM.tif]
